# Time trends in adult chronic disease inequalities by education in Brazil: 1998–2013

**DOI:** 10.1186/s12939-016-0426-5

**Published:** 2016-11-17

**Authors:** Hiram Beltrán-Sánchez, Flavia C. D. Andrade

**Affiliations:** 1 Department of Community Health Sciences at the Fielding School of Public Health and California Center for Population Research, University of California Los Angeles, 650 Charles E. Young Dr, 41-257 CHS, Box 951772, Los Angeles, CA 90095-1772 USA; 2 Department of Kinesiology and Community Health, University of Illinois at Urbana-Champaign, Champaign, USA

**Keywords:** Equity, Diabetes, Hypertension, Heart Disease, Education, Brazil

## Abstract

**Background:**

Socioeconomic differences in health in Brazil are largely driven by differences in educational attainment. In this paper, we assess whether educational gradients in chronic disease prevalence have narrowed in Brazil from 1998 to 2013, a period of a booming economy accompanied by major investments in public health in the country.

**Methods:**

Individual-level data came from the 1998, 2003 and 2008 Brazilian National Household Survey and the 2013 National Health Survey. We first evaluate age-standardized prevalence rates of chronic disease by education and second, we predict the estimated prevalence rate between those in low vs. high education to assess if relative changes in chronic disease have narrowed over time. Third, we estimate the slope index of inequality (SII) that evaluates the absolute change in the predicted prevalence of a disease between those in low vs. high education. Finally, we tested for statistically significant time trends in adult chronic disease inequalities by education.

**Results:**

Prevalence of diabetes and hypertension have increased over the period, whereas the prevalence of heart disease decreased. Brazilian adults with no education had higher levels of diabetes, hypertension and heart disease than those with some college or more. Adjusted prevalence for hypertension and heart disease indicate some progress in reducing educational disparities over time. However, for diabetes, adjusted results show a continuously increasing educational disparity from 1998 to 2013. By 2013, individuals with no education had about two times higher diabetes prevalence than those with higher education with larger disparity among women.

**Conclusions:**

Results confirm findings from previous work that educational inequalities in health are large in Brazil but also provide evidence suggesting some improvement in narrowing these differentials in recent times. Recent policies aiming at reducing the prevalence of obesity, smoking and alcohol consumption, and increasing physical activity and consumption of fruits and vegetables may increase the overall health and wellbeing of the Brazilian population. These programs are likely to be more effective if they target those with low socioeconomic status, as they appeared to be at a higher risk of developing chronic conditions, and promote educational opportunities.

## Background

During the past decades, Brazil has undergone major epidemiological and nutritional transitions. One major effect of these transitions has been an increase in the prevalence of chronic non-communicable diseases, particularly diabetes, hypertension and cardiovascular disease [1]. Estimates for 2013 indicate that Brazil has the fourth largest population of adults aged 20–79 with diabetes in the world (11.9 million; 9.0 % prevalence); this number is expected to rise to 19.2 million (11.7 %) by 2035 [2]. On the other hand, prevalence of hypertension has been declining in Brazil in the last decades. Estimates point to prevalence rates of 36.1 % in the 1980s and around 28.7 % in the 2000s [3]. Nonetheless, prevalence of hypertension remains high in Brazil affecting about one-fourth of all adults [3].

Chronic non-communicable diseases, such as diabetes, hypertension and coronary heart disease are associated with large financial burdens to national economies due to increase health costs and health care utilization that threaten the stability of public health system such as that in Brazil. Some evidence shows that ambulatory and hospitalization costs related to cardiovascular diseases and diabetes in Brazil are at about US 751 million and US 24 million in 2008–2010, respectively [4]. Most of the costs under cardiovascular disease are due to coronary heart disease, followed by cardiac failure and arterial hypertension [4]. The economic impact of chronic conditions extend beyond the medical costs, some estimates for 2008 indicate that the annual cost related to severe cardiovascular disease (i.e., require hospitalization at least once during the year) were mostly due to loss of productivity (55 %), followed by health care costs (36 %) and social security and employer’s reimbursement (8 %) [5]. Direct costs related to severe cardiovascular disease accounted for 8 % of the national expenditure on health [5].

Chronic non-communicable diseases are also responsible for a large mortality toll in the Brazilian adult population, in 2007, non-communicable diseases were responsible for about three-fourths (72 %) of all deaths [1]. Among these diseases, stroke and coronary heart disease are the main causes of death [6]. Nonetheless, some progress has been made in recent years as evidenced by the decline in age-standardized mortality rates due to chronic non-communicable diseases between 1996 and 2007 [1]. For example, mortality rates due to acute complications of diabetes declined between 1991 and 2010 [7]. Between 1996 and 2007, age-adjusted mortality attributable to non-communicable diseases declined by about 20 %, primarily associated with reductions in cardiovascular disease [1, 8]. However, these mortality improvements have not been equally experienced across populations’ subgroups. A study in São Paulo, for example, showed that even though reductions in heart disease mortality have been seen across all social groups, declines seemed more evident among wealthier segments of the society [9].

These socioeconomic differences in mortality, and health in particular, are so permissive and long enduring that reducing the inequalities “with a particular focus on the most vulnerable segments of society” is a major goal of Latin American countries as stated in the most recent PAHO Millennium Development Goals (http://www1.paho.org/english/mdg/cpo_pahoymdgs.asp). Importantly in Brazil, as in most countries, socioeconomic differences in health are largely driven by differences in educational attainment [10] whereby those with low levels of education tend to be in vulnerable positions leading to worse health outcomes relative to those with high education (this is commonly referred to as educational gradients in health). For instance, most studies in Brazil have found higher prevalence of diabetes among adults with lower education than among those with higher educational levels [11–15]. Goldenberg and colleagues found that whereas women that are more educated had lower prevalence of diabetes, the reverse was found among men in Sao Paulo [16]. Similarly, most studies in Brazil have identified an inverse association between education and prevalence of hypertension [17, 18] and heart disease (i.e. angina) [14]. However, Barreto and colleagues [19] reported no educational differences in hypertension after controlling for additional factors, such as age and sex.

An important limitation of the current Brazilian literature is that most studies addressing educational disparities on chronic disease (e.g., diabetes, hypertension and heart disease) are based on samples covering small geographical areas that are mostly urban [11–14, 16–18, 20, 21] which limits our understanding of population health in the country. Although there are few studies using nationally representative data, they all seem to suggest worse health outcomes among people with low educational levels. For example, one of the few studies using nationally representative data from the 2003 Brazilian World Health Survey found higher prevalence of diabetes and angina among those with incomplete primary schooling than those with complete ones [14]. More recently, based on nationally representative data from 2008, higher prevalence rates of hypertension were reported among men and women with lower education than among their counterparts with higher education [22]. However, because the educational classification used in these studies is not comparable, it is unclear if there have been improvements in chronic disease among those with low education and whether the educational gaps in chronic disease have narrowed or widened over the last years.

In this paper, we assess whether educational gradients in chronic disease prevalence have narrowed in Brazil from 1998 to 2013. We use three waves (1998, 2003, and 2008) of the Brazilian National Household Survey (Pesquisa Nacional por Amostra de Domicílios, PNAD) and the 2013 National Health Survey (Pesquisa Nacional de Saúde, PNS) to estimate disease prevalence in self-reported diabetes, hypertension and heart disease among adults aged 25–94. We estimate regression models controlling for age, education, race, region, and having health insurance to assess changes in educational gradients in chronic disease over time. First, we predict the estimated prevalence rate by educational level to assess if the gap in the predicted prevalence between those in low vs. high education has narrowed over time. Second, we estimate the slope index of inequality (SII) that evaluates the absolute change in the predicted prevalence of a disease between those in low vs. high education. We do this by survey-year and similarly assess if there have been significant changes in the SII over time. Our focus is to clarify whether trends in educational gradients in chronic disease prevalence have been reduced in a period of a booming economy accompanied by major investments in public health in Brazil.

## Methods

### Survey and setting

Individual-level data came from the 1998, 2003 and 2008 PNAD and the 2013 PNS. PNAD is a repeated cross-sectional in-person household survey that collects information on sociodemographic characteristics, such as education, work and earnings of the Brazilian population. In 1998, 2003 and 2008 the PNAD included a health component consisting of a series of questions on health conditions, disease diagnoses, and health care service utilization. A multistage, probability sampling design was adopted by the PNAD to produce national estimates pertaining to the Brazilian population. In the first stage, municipalities were selected at random. In the second stage, census tracts were randomly chosen from each selected municipality, with the inclusion probability proportional to the number of households in a census tract. In the third stage, households for interview were randomly chosen from each selected census tract. PNS is a household-based survey that collects information on health status and lifestyle of the Brazilian population, as well as access and use of health services. In addition, it also contains information on sociodemographic characteristics, such as educational attainment. A multistage, probability sampling design was adopted by the PNS to produce national estimates pertaining to the Brazilian population. The PNS sample is a subsample of the Brazilian Census Bureau (Instituto Brasileiro de Geografia e Estatistica, IBGE) master sample of the Sistema Integrado de Pesquisas Domiciliares (Integrated System of Household Surveys) which is constituted by the census tracks of the Brazilian 2010 census, except those that are very small or are considered special. The master sample is composed by a group of areas, which are considered primary sampling units (PSU), and the PNS sample was selected in three stages. In the first stage, the selection of the subsample of the PSU in each stratum of the master sample was proportional to the size. In the second stage, households were randomly sampled from the PSU selected in the first stage. In the third stage, one adult (18 years or older) was randomly chosen among all adults in the household [23]. The PNS questionnaire is divided into three parts. The first two parts are answered by one resident who provide information on the household characteristics and the health status of all household members. The last part is answered by the selected adult who provides information on the individual questionnaire, which included questions on chronic conditions, lifestyle, oral health, among others [23].

Detailed information about the PNAD and PNS including questionnaires, survey design, and datasets can be found in the Brazilian Census Bureau website (www.ibge.gov.br) and in the Fundação Instituto Oswaldo Cruz (Fiocruz) (www.pns.fiocruz.br). PNAD is conducted by the Brazilian Census Bureau. PNS is carried out by the Ministry of Health in partnership with the Brazilian Census Bureau. This paper we used PNAD and PNS de-identified public data and was deemed exempt from human subjects review.

### Participants

The 1998 PNAD interviewed 172,338 individuals 25–94 years of age from the 27 Brazilian states and the Federal District. In 2003, the sample was 202,069 and the 2008 PNAD interviewed 222,697 individuals. Among these respondents, 619, 1194 and 839 participants had missing values for the covariates of interest in 1998, 2003 and 2008, respectively. The final analytic sample based on the PNAD consisted of 594,452 adults with complete data on the variables of interest. The 2013 PNS interviewed 120,982 individuals 25–94 years of age, but morbidity data are only available for the selected adult (*N* = 52,323). However, final sample sizes vary by comorbidity: diabetes (*n* = 47,035), hypertension (*n* = 51,218) and heart disease (*n* = 52,323).

### Health outcomes

Health outcomes included previous diagnosis of hypertension, diabetes and heart disease. The questionnaire to ascertain these conditions was similar in 1998 and 2003 but it changed in the last two surveys. For example, in 1998 and 2003, the question was “…have [health condition]?” (…tem [doença]?” However, in 2008 the wording changed to “Has a doctor or a health professional ever told that you have [health condition]” (Algum médico ou profissional de saúde disse que tem [doença]). The wording in the PNS 2013 is somewhat similar to PNAD 2008 and reinforced the concept of medical diagnosis “Has a doctor has given you a diagnosis of [health condition]? (Algum médico já lhe deu o diagnóstico de [doença]?). Those who responded affirmatively in each survey-year were considered having the chronic condition and those who responded negatively as not having it. Women who reported having being diagnosed with diabetes or hypertension during pregnancy were classified as not having the condition. For additional details on question wording, see Appendix.

### Education

Four categorical variables for educational level (no education, primary, secondary and some college or more) were used to construct an education variable that was comparable between PNAD and PNS. No education corresponds to people who had no education or less than one year of formal schooling; ‘primary or secondary incomplete’ education are those with one to ten years of completed formal education; ‘secondary’ education are those who completed eleven years of schooling; and ‘some college or more’ are those who completed twelve or more years of schooling.

### Other individual characteristics

The following individual characteristics were controlled in regression analyses: a dichotomous variable for female (male as the reference group), a continuous variable for age in years; race (White versus non-White -- which included Black, Pardo, and those of Asian descent and indigenous); region of residence (South, Southeast, Midwest, Northeast, North); a dichotomous variable for proxy respondent; and a dichotomous variable for private health insurance. Those who reported having more than one health insurance were classified as having health insurance. See Appendix for details.

### Statistical analysis

Descriptive statistics for each survey year are presented in Table 1. Table 2 presents age-adjusted prevalence rates of chronic conditions based on the 2000 Brazilian census age distribution. We used two statistical approaches to further assess time trends in educational gradients in chronic disease. First we use multivariate logistic regression to examine the relationship between educational level and chronic disease prevalence, adjusting for age, sex, race, region of residence, health insurance and year. Next, we include an interaction term between education and survey-year to test whether the odds of reporting chronic conditions by educational groups differed over time. The estimations of interest are the effects of the education dummy variables on the log odds of an outcome. However, comparisons of estimated effects on log odds of prevalence for a health outcome across subsamples are influenced by the size of differentials in the levels of prevalence of the disease in each subsample. To circumvent this problem and to establish consistency with previous research, we compute ratios (R) of predicted prevalence of an outcome in the lowest education to the predicted prevalence in the highest education group.

**Table 1:** Descriptive statistics for the total population aged 20 or older (%), Brazil 1998–2013

Population characteristics	Survey-year			
	1998	2003	2008	2013
Education				
No education	18.72	16.03	13.72	15.42
Primary or incomplete secondary	58.89	55.73	50.71	41.05
Secondary	12.85	17.13	22.16	25.71
Some college or more	9.54	11.11	13.41	17.81
Age				
25–49	67.53	66.16	63.67	59.34
50–94	32.47	33.84	36.33	40.66
Sex				
Male	47.42	47.16	47.09	46.71
Female	52.58	52.84	52.91	53.29
Race				
White	57.45	54.89	50.79	48.23
Non-White	42.56	45.11	49.22	51.77
Proxy				
No	56.4	56.73	64.81	31.58
Yes	43.6	43.27	35.19	68.42
Region				
North	4.15	4.98	7.00	7.10
Northeast	26.05	25.92	25.67	26.18
Midwest	6.78	7.12	7.26	7.31
Southeast	46.86	46.22	44.97	44.44
South	16.17	15.76	15.10	14.97
Health Insurance				
No	72.01	71.88	71.02	68.61
Yes	27.99	28.12	28.98	31.99

All percentages shown are weighted taking into account the sampling complex design of each survey. Data for 1998–2008 come from PNAD and for 2013 from PNS

**Table 2:** Age-adjusted prevalence rates (%) of chronic conditions for the total population aged 25 or older by education, Brazil: 1998–2013

	Total	No education	Primary	Secondary	Some college or more	Ratio: No ed vs. Some college or more
Diabetes						
1998	3.72 (3.62, 3.83)	3.88 (3.65,4.12)	3.99 (3.84,4.14)	2.81 (2.48,3.13)	2.86 (2.52,3.20)	1.30
2003	4.52 (4.41, 4.64)	5.22 (4.93, 5.51)	4.70 (4.56, 4.85)	3.70 (3.43, 3.97)	3.47 (3.16, 3.79)	1.30
2008	5.54 (5.43, 5.65)	6.30 (5.99, 6.60)	5.93 (5.78. 6.09)	4.64 (4.40, 4.89)	4.12 (3.83, 4.40)	1.34
2013	6.76 (6.39, 7.13)	8.11 (7.03, 9.19)	7.70 (7.10, 8.30)	5.46 (4.71, 6.21)	4.58 (3.73, 5.42)	1.48
overall change	3.04	4.23	3.71	2.65	1.72	
Hypertension						
1998	19.94 (19.68, 20.20)	23.97 (23.33, 24.62)	20.33 (20.03,20.63)	15.70 (15.08, 16.31)	13.80 (13.14, 14.46)	1.44
2003	21.36 (21.12, 21.60)	25.36 (24.67, 26.04)	22.57 (22.28, 22.86)	16.18 (15.67, 16.70)	15.81 (15.22, 16.39)	1.35
2008	22.10 (21.88, 22.33)	26.22 (25.55, 26.89)	23.73 (23.44, 24.03)	18.12 (17.71, 18.53)	16.71 (16.20, 17.22)	1.32
2013	22.54 (21.93, 23.16)	25.28 (23.46, 27.10)	24.96 (23.96, 25.96)	19.81 (18.57, 21.04)	19.10 (17.62, 20.58)	1.18
overall change	2.60	1.31	4.63	4.11	5.30	
Heart problems						
1998	6.95 (6.78, 7.11)	8.26 (7.88,8.63)	7.07 (6.87,7.27)	5.60 (5.17,6.04)	4.24 (3.84,4.65)	1.64
2003	6.13 (5.99, 6.26)	7.03 (6.70, 7.36)	6.50 (6.33, 6.68)	4.39 (4.10, 4.69)	4.53 (4.18, 4.89)	1.35
2008	5.82 (5.70, 5.94)	6.76 (6.44, 7.09)	6.26 (6.10, 6.42)	4.60 (4.36, 4.84)	4.10 (3.83, 4.38)	1.42
2013	4.20 (3.87, 4.53)	4.82 (4.00, 5.63)	4.70 (4.18, 5.23)	3.38 (2.71, 4.05)	3.43 (2.55, 4.31)	1.22
overall change	−2.75	−3.44	−2.37	−2.22	−0.81	

Prevalence rates have been adjusted using the Brazilian total population in year 2000 as the standard

Second, we estimate the slope index of inequality (SII) by survey-year and similarly assess if there have been changes in the SII over time. The SII evaluates the absolute change in the predicted prevalence of a disease between those in low vs. high education controlling for the changing distribution of different people in the education distribution [24]. We estimate the SII through a regression-based method including controls for age, sex, race, region of residence, and health insurance. The SII has been widely used in studies of health and mortality inequalities in several countries [25].

**Fig. 1:**
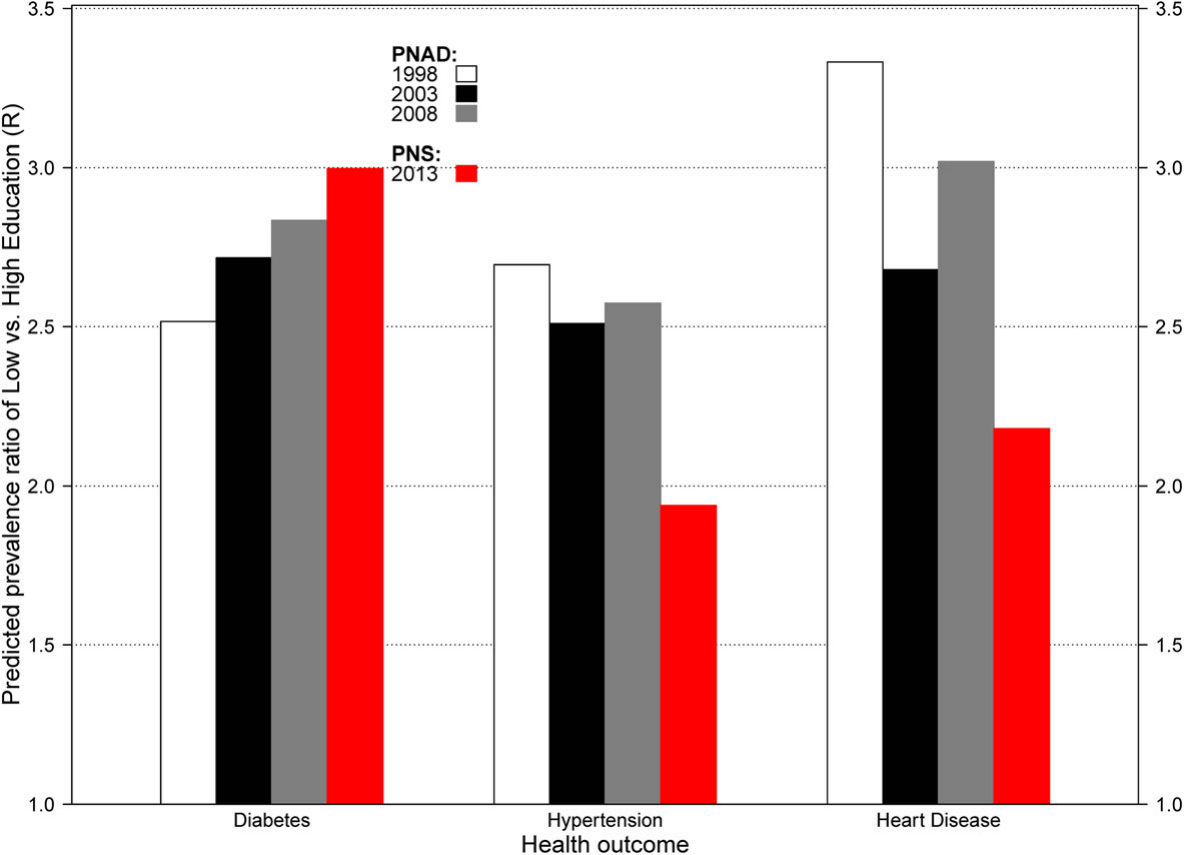
Predicted prevalence ratio of an outcome in the lowest education to the predicted prevalence in the highest education group (R) for the total population, Brazil 1998–2013. Note: Predicted prevalence rates estimated from models in Table 3

These analyses allow us to assess if there has been a reduction in the gap in absolute terms, through SII, and in relative terms as shown in Fig. 1, in the prevalence of chronic conditions between people with low and high education. Both descriptive statistics and regression analyses accounted for multistage probability sampling design. Statistical analyses were performed in Stata 12.1 SE version (StataCorp, College Station, TX). Given the high proportion of responses provided in the PNAD by proxy respondents, which may differ from self-reports,[26] further sensitivity analyses excluded data provided by proxy respondents and yielded similar substantive results.

## Results

Table 1 provides descriptive statistics for the four survey-years. Results show educational improvements during the 1998–2013 period. In particular, the proportion of people with some college or more almost doubled between 1988 and 2013, from 9.54 % in 1998 to 17.81 % in 2013, while the proportion of those with no education declined by about 3 percentage points, from 18.8 % in 1998 to 15.4 % in 2013.

Age-adjusted prevalence of diabetes, hypertension and heart problems by education and survey-year is shown in Table 2. Results indicate that while the prevalence of diabetes and hypertension have been on the rise, the prevalence of heart disease is actually declining. The prevalence of diabetes and hypertension for the total population increased from 3.72 to 6.76 % and from 19.94 to 22.54 %, respectively, in the period 1998 to 2013. Heart problems, on the other hand, decreased from 6.95 to 4.20 %. Moreover, there was a substantial educational gradient across all chronic conditions with higher prevalence among those with no education than among those with some college or more.

Moreover, the differential trend in the educational gradient in the prevalence of hypertension, heart and diabetes resulted from a mix time trends in the underlying prevalence of these conditions by education (Table 2). For hypertension, for example, the reduction in the educational gradient between 1998 and 2013 resulted from faster rise in the prevalence among those with some college or more. In contrast, the reduction in the educational gradient for heart disease is due to faster lowering of the prevalence among those with no education. On the contrary, the increase in the educational gradient in diabetes over time is the result of faster rise in the prevalence among people with no education.

Tables 3 presents the results of education from logistic regressions for diabetes, hypertension and heart disease. Results based on both sexes indicate statistically significant educational gradients for all chronic diseases in which those with more education were less likely to report having any of these conditions. Results also indicate significant increases in the prevalence of diabetes and hypertension over time, but significant decreases in heart disease prevalence. When analyses are disaggregated by sex, results further elucidate that the educational gradients for the total population are more strongly concentrated among women. A statistically significant educational gradient was observed among women, but among men, higher prevalence of chronic conditions is mostly observed among those with less than secondary education. No statistical differences were found between men with secondary and those with more than secondary education. For both men and women, prevalence of diabetes and hypertension increased over time, while prevalence of heart disease decreased.

**Table 3:** Odds ratios from logistic regressions for Diabetes, Hypertension and Heart Disease for the total population aged 25 or older and by gender, Brazil 1998–2013

Variables	All			Females			Males		
	Diabetes	Hypertension	Heart	Diabetes	Hypertension	Heart	Diabetes	Hypertension	Heart
Education									
No education	2.07***	1.99***	2.17***	3.36***	2.87***	3.06***	1.12	1.25***	1.51***
Primary or incomplete secondary	1.81***	1.68***	1.72***	2.56***	2.30***	2.30***	1.28***	1.14***	1.29***
Secondary	1.21***	1.11***	1.15**	1.37***	1.29***	1.41***	1.13	0.93	0.96
Some college or more	1.00	1.00	1.00	1.00	1.00	1.00	1.00	1.00	1.00
Year									
1998	1.00	1.00	1.00	1.00	1.00	1.00	1.00	1.00	1.00
2003	1.27***	1.14***	0.89***	1.24***	1.15***	0.88***	1.31***	1.12***	0.93***
2008	1.63***	1.24***	0.87***	1.56***	1.22***	0.82***	1.77***	1.28***	0.95*
2013	2.14***	1.36***	0.64***	2.10***	1.36***	0.59***	2.22***	1.37***	0.72***

Models control for age, sex (for all), race, region, health insurance and proxy respondent (see Appendix for full tables)

*** *p* <0.001, ** *p* <0.01, * *p* <0.05

Next, we evaluate whether educational differentials significantly changed over time by including an interaction term for education and year (Table 4). Results confirm higher levels of disease prevalence among those with less than secondary education in recent years compared to those with some college or more, except for diabetes among males with no education and for heart disease. Results also confirm the increase in diabetes and hypertension prevalence over the years. On the other hand, for heart disease there are no statistically significant time trends. Education and year interaction terms indicate faster increases in diabetes prevalence among those with less than some college compared to those with some college or more, but results are mostly driven by men. On the other hand, those with less than some college seem to have slower rates of increase on the prevalence of hypertension.

**Table 4:** Odd ratios from logistic regressions including an Education-Year interaction term for Diabetes, Hypertension and Heart Disease for the Total Population and by Sex, Brazil 1998–2013

Variables	All			Females			Males		
	Diabetes	Hypertension	Heart	Diabetes	Hypertension	Heart	Diabetes	Hypertension	Heart
Education									
No education	1.66***	2.22***	2.70***	2.83***	3.34***	3.58***	0.82*	1.40***	1.98***
Primary or incomplete secondary	1.60***	1.76***	1.97***	2.32***	2.52***	2.47***	1.17*	1.20***	1.58***
Secondary	0.99	1.17***	1.40***	1.08	1.44***	1.57***	1.01	1.00	1.31***
Some college or more	1.00	1.00	1.00	1.00	1.00	1.00	1.00	1.00	1.00
Year									
1998	1.00	1.00	1.00	1.00	1.00	1.00	1.00	1.00	1.00
2003	1.20**	1.16***	1.05	1.14	1.25***	1.03	1.31***	1.12**	1.11
2008	1.48***	1.22***	0.94	1.37***	1.29***	0.89	1.70***	1.23***	1.05
2013	1.70***	1.55***	0.84	1.80***	1.58***	0.65**	1.74***	1.62***	1.07
Interaction education X year									
No education									
2003	1.15	0.98	0.81***	1.17	0.92	0.80**	1.19	0.99	0.81**
2008	1.19**	1.03	0.90	1.21	0.95	0.91	1.26**	1.07	0.89
2013	1.39**	0.74***	0.64***	1.22	0.73***	0.76	1.63**	0.71***	0.56**
Primary or incomplete secondary									
2003	1.00	1.01	0.87*	1.04	0.94	0.89	0.94	1.02	0.84*
2008	1.05	1.03	0.94	1.09	0.95	0.94	0.99	1.06	0.93
2013	1.24*	0.89*	0.79	1.14	0.87	0.94	1.25	0.85	0.68*
Secondary									
2003	1.15	0.90**	0.74***	1.23	0.83***	0.72***	1.04	0.94	0.74**
2008	1.19*	0.99	0.87*	1.33*	0.92	0.90	1.00	0.99	0.82*
2013	1.32*	0.92	0.77	1.29	0.90	0.98	1.27	0.88	0.60**

Models were fitted separately by chronic disease controlling for sex (for all), race, region, health insurance and proxy respondent (see Appendix for full tables)

*** *p* <0.001, ** *p* <0.01, * *p* <0.05

Results for the slope index of inequality (SII) show similar results regarding time trends in educational gradients in chronic disease (Table 5). This table shows coefficient estimates for the log-odds of self-reporting a chronic disease; negative numbers in the SII indicate that people with high education have lower likelihood than those with low education of having a chronic disease indicating an inequality in the condition. Coefficient estimates for the interactions of SII and year show statistically significant increases over time (the interactions are negative) in the SII for diabetes and hypertension but not for heart disease. Importantly, there is a growing inequality over time for diabetes (the SII-year interaction becomes more negative in recent times) but a reduction in the inequality by 2013 for hypertension (positive interaction coefficient). These results hold for both men and women, although the interaction of SII and year for hypertension is not statistically significant by 2013 among females.

**Table 5:** Log-odds from logistic regressions estimating the slope inequality index (SII) and its interaction with time for Diabetes, Hypertension and Heart Disease for the Total Population and by Sex, Brazil 1998–2013

Variables	All			Females			Males		
	Diabetes	Hypertension	Heart	Diabetes	Hypertension	Heart	Diabetes	Hypertension	Heart
Slope inequality index (SII)	−0.55***	−0.87***	−0.99***	−1.01***	−1.17***	−1.19***	0.24**	−0.42***	−0.68***
Year									
1998	1.00	1.00	1.00	1.00	1.00	1.00	1.00	1.00	1.00
2003	0.29***	0.14***	−0.16***	0.27***	0.16***	−0.17***	0.37***	0.12***	−0.13**
2008	0.54***	0.21***	−0.19***	0.49***	0.20***	−0.23***	0.72***	0.27***	−0.08
2013	0.88***	0.15***	−0.63***	0.80***	0.16***	−0.68***	1.05***	0.13**	−0.56***
Interaction SII X year									
2003	−0.18**	−0.08*	0.03	−0.23**	−0.13***	−0.00	−0.21*	−0.04	0.06
2008	−0.25***	−0.13***	−0.04	−0.33***	−0.19***	−0.10	−0.33***	−0.12*	−0.02
2013	−0.48***	0.16**	0.24	−0.50***	0.01	0.08	−0.59***	0.32***	0.39

Models were fitted separately by chronic disease controlling for sex (for all), race, region, health insurance and proxy respondent (see Appendix for full tables)

*** *p* <0.001, ** *p* <0.01, * *p* <0.05

**Fig. 2:**
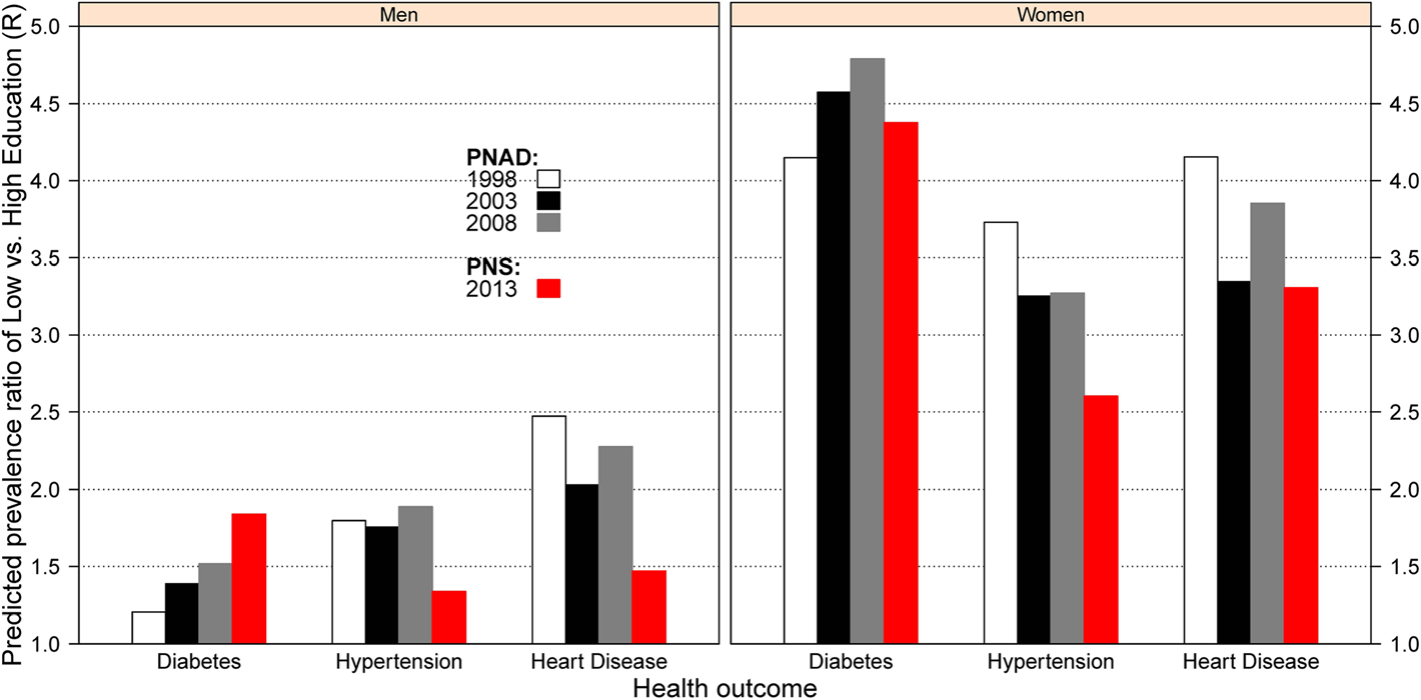
Predicted prevalence ratio of an outcome in the lowest education to the predicted prevalence in the highest education group (R) for Men and Women, Brazil 1998–2013. Note: Predicted prevalence rates estimated from models in Table 3

To further elucidate the educational gradients in chronic disease, we use coefficient estimates from Table 3 to compute the predicted prevalence ratio of an outcome in the lowest education to the predicted prevalence in the highest education group (Fig. 1). This is a measure of the relative change in the educational gradient in chronic disease. The educational gradient holds for all the observed years indicating a persistent disparity in health. For example, there is a continuously increasing educational disparity in diabetes prevalence from 1998 to 2013 in which individuals with low education had about 2 times higher prevalence by 2013 than those with high education. However, results for hypertension and heart disease indicate some progress in reducing educational disparities in chronic disease over time, although adults with low education still experienced higher prevalence of these conditions by 2013. Results from Table 3 translate into predicted prevalence ratios that indicate larger educational gradients in chronic disease among women than men (Fig. 2).

Among women, educational gradients in diabetes reached very large levels in which women with no education experienced about 3 times higher prevalence than their counterparts with high education. Importantly, educational gradients for hypertension and heart disease were reduced by 2013, although the magnitude of these differences remains higher than those observed among men. The women educational gradient in diabetes increased from 1998 to 2008, but decreased in the most recent period. Among men, educational gradients in hypertension and heart disease were also reduced by 2013. The men educational gradient in diabetes continuously increased over time and by 2013 the gradient had widened even more so that men with no education had about 30 % higher prevalence than those with high education.

Because educational attainment differs by age and sex, we ran additional regressions to evaluate whether educational differentials in chronic disease operated differently among older and younger men and women (see Appendix). For women, the educational gradient in all chronic conditions remains statistically significant for younger and older adults across all years. The only exception is that young and older women with secondary education did not differ from those with some college or more on their odds of reporting diabetes. For younger and older men, those with less than some college had higher odds of reporting having hypertension and heart disease. The only exception was among older men with secondary education that did not differ from those with college in reporting hypertension. For diabetes, the only statistical significance was found among those with primary or incomplete secondary that had higher odds of reporting diabetes than those with some college or more.

## Discussion

This study examined the educational inequalities in the prevalence of diabetes, hypertension and heart disease between 1998 and 2013. We found that the adjusted prevalence of diabetes and hypertension increased over the period 1998–2013, whereas the prevalence of heart disease decreased, controlling for demographic and socioeconomic factors. These results confirm findings from a previous study based on older adults in Brazil, which also used the PNAD 1998, 2003 and 2008 [27]. We also found that educational inequalities in diabetes prevalence have widen from 1998 to 2013, but for hypertension and heart disease have narrowed. Brazilian adults with no education had higher levels of diabetes, hypertension and heart disease than those with some college or more. Educational inequalities were higher among women. These results confirm findings from previous work that educational inequalities in health are large in Brazil but also provide evidence suggesting some improvement in narrowing these differentials in recent times.

Brazil is marked by socioeconomic inequalities that influence health status. For all periods, diabetes was more prevalent among individuals with lower educational levels. This finding is similar to previous studies that identified higher levels of diabetes, hypertension and heart disease among those with lower educational levels in Brazil [11–14, 28]. For example, Lima-Costa and colleagues based on adults aged 50 and over in Brazil found that prevalence of diabetes was 20 % higher among those with 8 or fewer years of schooling versus those with twelve or more [28]. Nonetheless, our results further elucidate different trends in educational gradients by sex. For diabetes prevalence, for example, while females experienced a reduction in the educational gap between 2008 and 2013, males’ educational gradient continuously increased. Our results also point to educational disparities in hypertension, which confirm previous studies in Brazil that found higher levels of hypertension among those with less education [18, 21, 28, 29]. Longo and colleagues, based on a sample of adults 20 to 59 years old in Lages, Southern Brazil, found that those with 0–4 years of schooling had prevalence rates 70 % higher than those with 12 or more years of schooling [18]. Lima-Costa and colleagues report that prevalence of hypertension was 20 % higher among those with 12 or more years of schooling than those with eight or fewer [28]. Educational gradients in hypertension prevalence rates have been observed in samples with self-reported and measured blood pressure [29], with some highlighting stronger educational disparities on hypertension prevalence among women than men [30]. Other cardiovascular risks are also more prevalent among individuals with lower education in Brazil [31], so it is not surprising the educational disparities we found in heart disease.

Our results also suggest a widening of the educational disparities in diabetes between 1998 and 2013. We found that the age-adjusted prevalence of diabetes increased at a faster pace among people with no education (from 3.9 % in 1998 to 8.1 % in 2013) than among those with some college or more (from 2.9 % in 1998 to 4.6 % in 2013) leading to a larger relative increase in the prevalence ratio. These findings may be associated with continued increases in the prevalence of obesity in Brazil, particularly among the poor [32]. Prevalence of obesity tripled among men in the last three decades, from 2.7 % in 1975 to 8.8 % in 2003, while among women, it almost doubled from 7.4 to 13.0 %, respectively [32]. Moreover, obesity prevalence continues to increase in recent years [33]. However, increases were not equally distributed across social groups and higher increases were seen among adults in the lowest income quintile [32]. Data from the Surveillance System of Risk and Protective Factors for Chronic Non-Communicable Diseases through Telephone Interviews (VIGITEL) on a sample of adults living in all state capitals in Brazil indicated that 10.8 % of adults were obese in 2006, with this percentage reaching 13.5 % in 2009 [34]. Among women, higher rates of obesity were not only higher among those with less education, but increases over time were more marked among those with less than 12 years of schooling [34]. On the other hand, obesity prevalence rates were found to be higher among more educated men in urban areas and faster increases were also found in this group [34]. The increase in obesity in Brazil will have an effect on the prevalence of diabetes and other obesity-related diseases in coming decades. If obesity prevalence continues to increase as predicted by past patterns, the number of cases of diabetes is expected to double between 2010 and 2050 [35]. However, even if interventions are successful at reducing people’s body mass index by 5 %, the number of cases would likely increase by 59 % [35]. In any case, the economic costs associated with diabetes are expected to increase in coming decades, but the economic and social burden may be lower if additional preventive and treatment care are available [36].

Prevalence of hypertension is also expected to continue an upward trend in the coming decades given the rise on obesity [35]. According to data from the Global Burden of Disease, low intake of fruits and whole grains and high intake of sodium were the main individual factors associated with cardiometabolic deaths in Brazil [37]. Suboptimal diet along with high systolic blood pressure are the main contributors to cardiometabolic deaths in Brazil [37]. Nonetheless, we found signs of improvement in reducing the prevalence of heart disease with no statistically significant changes over time in the slope inequality index. In addition, we also highlight progress in reducing the educational gradient in hypertension and heart disease from 1998 to 2013. These improvements in cardiovascular disease are likely the result of public health interventions such as the Family Health Program (FHP) and of tobacco control programs aimed at reducing smoking prevalence. For example, the FHP has been associated with reductions in cardiovascular morbidity and mortality across municipalities in Brazil between 2000 and 2009 [38] and with reductions in chronic disease hospitalization rates from 1999 to 2007 including hypertension, stroke and other CVD conditions [39]. Importantly, municipalities with the highest rates of enrollment in the FHP are characterized by having small populations with very high levels of illiteracy [39]; it is thus likely that people with low levels of education could have experienced major improvements in cardiovascular outcomes. This is consistent with our findings of faster reductions in adjusted heart disease prevalence among people with low education (8.3 % in 1998 to 4.8 % in 2013) than among those with high education (4.2 % in 1998 to 3.4 % in 2013) which led to reductions in the educational gradient in heart disease by 2013 (Fig. 1). In addition, some evidence indicates that smoking prevalence in Brazil was reduced by about half in just two decades, from 32 % in 1989 to 17 % in 2008, and the decline occurred across all educational levels [40]. These reductions in smoking prevalence have been linked to implementation of programs to increase cigarette taxes, smoke-free air policies, mass media campaigns, marketing bans, and cessation treatment programs, among others [41].

A few limitations of this study should be noted. Health conditions were reported by either the respondent or proxy respondent, which may be subject to diagnosis bias and avoidance of diagnosis [42]. Even when reported by the participant, there is evidence that self-reports may not be in agreement with clinical measurements. Previous studies in Brazil have shown that individuals tend to overreport having hypertension [29]. Diabetes, on the other hand, tends to be underreported which suggests that our results provide a lower bound of the diabetes burden in Brazil. For instance, data from the Brazilian Longitudinal Study of Adult Health, a cohort study of adult civil servants aged 35–74 years found that 50.4 % of the individuals with diabetes were undiagnosed [15]. In addition, accuracy of self-reports may also vary depending on socioeconomic characteristics, such as educational level and access to health insurance. For instance, it is possible that those with higher education and access to health insurance be more aware of their health [42]. However, if this were to be the case we would have expected higher prevalence among people with high education; the fact that we observed the opposite indicates that this issue is unlikely to explain our estimates of the educational gradients in chronic disease over time. It is also possible that those with lower education (and possibly lower health literacy) to have more difficulty understanding the health diagnosis and answering the survey questions [42].

Bias may also arise based on avoidance of diagnosis, which can differ across groups. Even though we do not have access to clinical data to confirm the validity of the self-reported data for all these years, we tested (analyses not shown) whether reporting of chronic conditions varied across educational levels for those with and without health insurance by adding an interaction between education and access to health insurance. Results indicate that once interactions are included, the educational gradient remains with those with lower education reporting higher prevalence of chronic conditions. For diabetes and heart disease, none of the education and health insurance interaction terms were statistically significant. For hypertension, those with lower education (i.e. less than secondary) and with health insurance were more likely to report having the condition than their counterparts without health insurance. Further analyses (not shown) indicate that access to health insurance increased during this period among those with no education, even though access remains more limited among those with lower education in Brazil.

Another limitation refers to the large share of the data in the PNAD that were provided by proxy respondents. Previous studies in Brazil discussed the validity of the information provided by proxy respondents, particularly regarding self-reported health [26]. Many studies have included a dummy variable in multivariate analyses to address this problem [43, 44]. In this study, we adopted two alternative strategies to assess proxy’s health status and their impact on our findings. The first approach was to include a dummy variable in the regression models and results indicated that proxy respondents tended to underreport hypertension among men and women and heart disease among men (see Appendix). Our second approach was to exclude individuals whose data were provided by proxy respondents and substantive results remained unchanged. It is thus unlikely that proxy respondents could bias our findings regarding the trends in educational gradients in chronic disease.

Changes in the questionnaire wording also raises some challenges about comparability of chronic disease prevalence over time. In 1998 and 2003, the PNAD inquired whether individuals had the chronic condition regardless of whether it had been diagnosed by a medical professional, but in the 2008 PNAD and the 2013 PNS the wording changed to emphasize medical diagnosis. In 2008 the respondent was asked if a physician or health professional had told him/her that he/she had the chronic condition whereas in 2013 the participant was asked if he/she had received a diagnosis from a physician. For heart disease, the change in the wording was even more pronounced in 2013 because the interviewers provided examples, such as heart attack, angina, and cardiac insufficiency, to clarify the meaning of heart diseases. These changes in the questionnaire can influence prevalence trends and may modify the association between education and self-reported chronic disease prevalence if knowledge and access to health care vary by education over time.

## Conclusion

In conclusion, we provided a more comprehensive picture of population health as well as some hints of changes in educational inequalities in chronic disease in Brazil between 1998 and 2013. These inequalities are more pervasive among women than men, and they clearly indicate that Brazilian adults with no education consistently experience higher levels of diabetes, hypertension and heart disease than those with some college or more. Nonetheless, there are signs of hope as the Brazilian government recently launched the Brazilian Strategic Action Plan to Combat Chronic Non-communicable Diseases, which aims at reducing the prevalence of obesity, smoking and alcohol consumption, and increasing physical activity and consumption of fruits and vegetables [45]. These efforts, if well implemented, may help reduce the growth of obesity, diabetes and hypertension in the country and increase the overall health and wellbeing of the population. Nonetheless, given the unequal distribution of resources in the Brazilian society, these programs are likely to be more effective if they target those with low socioeconomic status (e.g., low education) as they appear to be at a higher risk of developing chronic conditions and promote educational opportunities.

## Appendix

**Table 6:** Description of variables used in the paper, 1998–2013

Variable	PNAD 1998	PNAD 2003	PNAD 2008	PNS 2013
State	UF	UF	UF	V0001
Sex	V0302 - Sexo	V0302 - Sexo	V0302 - Sexo	C006 – Sexo
Age	V8005_idade	V8005 – Idade do morador na data de referencia	V8005 – Idade do morador na data de referencia	C008 - Idade
Race	A cor ou raça do (a) é	V0404 - Cor ou raça	V0404 - Cor ou raça	C009 - Cor ou raça
Diabetes	V1312 – … tem diabetes?	V1312 – tem diabetes	V1312 - Algum médico ou profissional de saúde disse que tem diabetes	Q030 - Algum médico já lhe deu o diagnóstico de diabetes?
Hypertension	V1314 - …tem hipertensão (pressão alta)?	V1314 - Tem hipertensão	V1314 - Algum médico ou profissional de saúde disse que tem hipertensão	Q002 - Algum médico já lhe deu o diagnóstico de hipertensão arterial (pressão alta)?
Heart disease	V1315 - …tem doença do coração?	V1315 - Tem doença do coração	V1315 - Algum médico ou profissional de saúde disse que tem doença do coração	Q063- Algum médico já lhe deu o diagnóstico de uma doença do coração tais como infarto, angina, insuficiência cardíaca ou outra?
Proxy	V1301 - O informante desta parte é	V1301 - O informante desta parte é	V1301 - O informante desta parte foi	J060 - O informante desta parte foi:
Health insurance	V1321 - Tem direito a algum plano de saúde (médico ou odontológico) particular, de empresa ou órgão público?	V1321 - Tem plano de saúde	V1321 - Tem direito a algum plano de saúde, médico ou odontológico, particular, de empresa ou órgão público	I001 - tem algum plano de saúde, médico ou odontológico, particular, de empresa ou órgão público?
Education	V4703_anos_estudos	V4703_anos_estudos	V4803_anos_estudos	VDD004

**Table 7:** Odds ratios from logistic regressions for Diabetes, Hypertension and Heart Disease for the total population aged 25 or older and by gender, Brazil 1998–2013

Variables	All			Females			Males		
	Diabetes	Hypertension	Heart	Diabetes	Hypertension	Heart	Diabetes	Hypertension	Heart
Education									
No education	2.07***	1.99***	2.17***	3.36***	2.87***	3.06***	1.12	1.25***	1.51***
Primary or incomplete secondary	1.81***	1.68***	1.72***	2.56***	2.30***	2.30***	1.28***	1.14***	1.29***
Secondary	1.21***	1.11***	1.15**	1.37***	1.29***	1.41***	1.13	0.93	0.96
Some college or more	1.00	1.00	1.00	1.00	1.00	1.00	1.00	1.00	1.00
Age									
25–49	1.00	1.00	1.00	1.00	1.00	1.00	1.00	1.00	1.00
50–94	5.62***	4.94***	4.70***	5.21***	5.02***	4.10***	6.14***	4.83***	5.62***
Sex									
Male	1.00	1.00	1.00	na	na	na	na	na	na
Female	1.27***	1.54***	1.24***						
Race									
White	1.02	0.89***	1.03	1.02	0.85***	0.98	1.02	0.95**	1.10**
Non-White	1.00	1.00	1.00	1.00	1.00	1.00	1.00	1.00	1.00
Proxy									
No	1.00	1.00	1.00	1.00	1.00	1.00	1.00	1.00	1.00
Yes	0.96	0.90***	0.90***	0.95	0.89***	0.93**	0.97	0.93***	0.89***
Region									
North	0.79***	0.69***	0.71***	0.80***	0.69***	0.72***	0.78***	0.70***	0.72***
Northeast	0.75***	0.80***	0.63***	0.79***	0.83***	0.62***	0.71***	0.77***	0.67***
Midwest	0.92**	0.94***	1.08**	0.97	0.95*	1.13***	0.87**	0.94*	1.02
Southeast	1.00	1.00	1.00	1.00	1.00	1.00	1.00	1.00	1.00
South	0.90***	0.97	1.16***	0.93	0.99	1.19***	0.85**	0.95	1.11**
Health Insurance									
No	1.00	1.00	1.00	1.00	1.00	1.00	1.00	1.00	1.00
Yes	1.20***	1.13***	1.19***	1.12***	1.07***	1.15***	1.32***	1.22***	1.26***
Year									
1998	1.00	1.00	1.00	1.00	1.00	1.00	1.00	1.00	1.00
2003	1.27***	1.14***	0.89***	1.24***	1.15***	0.88***	1.31***	1.12***	0.93***
2008	1.63***	1.24***	0.87***	1.56***	1.22***	0.82***	1.77***	1.28***	0.95*
2013	2.14***	1.36***	0.64***	2.10***	1.36***	0.59***	2.22***	1.37***	0.72***

*** *p* <0.001, ** *p* <0.01, * *p* <0.05

**Table 8:** Logistic regression results including an Education-Year interaction term for Diabetes, Hypertension and Heart Disease for the Total Population and by Sex, Brazil 1998–2013

Variables	All			Females			Males		
	Diabetes	Hypertension	Heart	Diabetes	Hypertension	Heart	Diabetes	Hypertension	Heart
Education									
No education	1.66***	2.22***	2.70***	2.83***	3.34***	3.58***	0.82*	1.40***	1.98***
Primary or incomplete secondary	1.60***	1.76***	1.97***	2.32***	2.52***	2.47***	1.17*	1.20***	1.58***
Secondary	0.99	1.17***	1.40***	1.08	1.44***	1.57***	1.01	1.00	1.31***
Some college or more	1.00	1.00	1.00	1.00	1.00	1.00	1.00	1.00	1.00
Year									
1998	1.00	1.00	1.00	1.00	1.00	1.00	1.00	1.00	1.00
2003	1.20**	1.16***	1.05	1.14	1.25***	1.03	1.31***	1.12**	1.11
2008	1.48***	1.22***	0.94	1.37***	1.29***	0.89	1.70***	1.23***	1.05
2013	1.70***	1.55***	0.84	1.80***	1.58***	0.65**	1.74***	1.62***	1.07
Interaction education x year									
No education									
2003	1.15	0.98	0.81***	1.17	0.92	0.80**	1.19	0.99	0.81**
2008	1.19**	1.03	0.90	1.21	0.95	0.91	1.26**	1.07	0.89
2013	1.39**	0.74***	0.64***	1.22	0.73***	0.76	1.63**	0.71***	0.56**
Primary or incomplete secondary									
2003	1.00	1.01	0.87*	1.04	0.94	0.89	0.94	1.02	0.84*
2008	1.05	1.03	0.94	1.09	0.95	0.94	0.99	1.06	0.93
2013	1.24*	0.89*	0.79	1.14	0.87	0.94	1.25	0.85	0.68*
Secondary									
2003	1.15	0.90**	0.74***	1.23	0.83***	0.72***	1.04	0.94	0.74**
2008	1.19*	0.99	0.87*	1.33*	0.92	0.90	1.00	0.99	0.82*
2013	1.32*	0.92	0.77	1.29	0.90	0.98	1.27	0.88	0.60**
Age (ref = ages 25–49)									
50–94	5.63***	4.93***	4.68***	5.21***	4.83***	5.61***	6.14***	4.83***	5.61***
Sex (ref = male)									
Female	1.27***	1.54***	1.24***	na	na	na	na	na	na
Race (ref = non-white)									
White	1.02	0.89***	1.03	1.01	0.95**	1.10**	1.01	0.95**	1.10**
Proxy									
Yes	0.95	0.90***	0.90***	0.97	0.93***	0.89***	0.97	0.93***	0.89***
Region (ref = Southeast)									
North	0.79***	0.69***	0.72***	0.78***	0.70***	0.72***	0.78***	0.70***	0.72***
Northeast	0.75***	0.80***	0.63***	0.71***	0.77***	0.67***	0.71***	0.77***	0.67***
Midwest	0.92**	0.94***	1.08**	0.87**	0.94*	1.02	0.87**	0.94*	1.02
South	0.90***	0.97	1.16***	0.85**	0.95	1.11**	0.85**	0.95	1.11**
Health Insurance									
Yes	1.20***	1.14***	1.20***	1.31***	1.23***	1.26***	1.31***	1.23***	1.26***

*** *p* <0.001, ** *p* <0.01, * *p* <0.05

**Table 9:** Odds ratios from logistic regressions for Diabetes, Hypertension and Heart Disease for Men and Women for younger adults (25–49 years), Brazil 1998–2013

Variables	Females			Males		
	Diabetes	Hypertension	Heart	Diabetes	Hypertension	Heart
Education						
No education	3.75***	4.09***	3.51***	0.84	1.23***	1.52***
Primary or incomplete secondary	2.41***	2.63***	2.16***	1.08	1.20***	1.32**
Secondary	1.02	1.38***	1.43***	1.01	1.04	1.21
Some college or more	1.00	1.00	1.00	1.00	1.00	1.00
Year						
1998	1.00	1.00	1.00	1.00	1.00	1.00
2003	1.11	1.18**	1.07	1.04	1.09	0.92
2008	1.64***	1.16**	0.85	1.66***	1.09	0.87
2013	2.00***	1.57***	0.78	1.43	1.58***	0.97
Interaction education x year						
No education						
2003	1.20	0.88	0.72**	1.37	0.93	0.89
2008	0.89	0.86*	0.78	1.02	0.94	0.79
2013	0.77	0.55***	0.90	1.70	0.79	0.46*
Primary or incomplete secondary						
2003	1.04	0.93	0.83	1.08	0.97	0.93
2008	0.94	0.94	0.97	0.91	1.01	1.04
2013	1.26	0.88	1.00	1.16	0.75**	0.74
Secondary						
2003	1.38	0.88	0.68***	1.27	0.95	0.91
2008	1.09	1.01	0.89	0.91	1.05	0.95
2013	1.47	0.90	1.04	1.49	0.78	0.63
Race (ref = white)						
Non-White	0.97	0.82***	0.89**	0.90	0.90***	0.89*
Proxy						
Yes	0.97	0.91***	0.86***	0.99	0.98	0.97
Region (ref = Southeast)						
North	0.67***	0.65***	0.75***	0.89	0.77***	0.88
Northeast	0.65***	0.79***	0.61***	0.73***	0.84***	0.71***
Midwest	0.99	0.91***	1.00	0.93	1.04	1.12
South	0.98	0.96	0.99	0.97	1.05	1.22**
Health Insurance						
Yes	1.08	0.98	1.03	1.15	1.16***	0.93

*** *p* <0.001, ** *p* <0.01, * *p* <0.05

**Table 10:** Odds ratios from logistic regressions for Diabetes, Hypertension and Heart Disease for Men and Women for older adults (50–94 years), Brazil 1998–2013

Variables	Females			Males		
	Diabetes	Hypertension	Heart	Diabetes	Hypertension	Heart
Education						
No education	1.94***	2.31***	2.56***	0.64***	1.11	1.46***
Primary or incomplete secondary	1.91***	2.02***	2.19***	1.10	1.10	1.43***
Secondary	1.08	1.44***	1.57***	1.06	1.05	1.40***
Some college or more	1.00	1.00	1.00	1.00	1.00	1.00
Year						
1998	1.00	1.00	1.00	1.00	1.00	1.00
2003	1.13	1.32***	0.99	1.48***	1.20**	1.22*
2008	1.16	1.42***	0.90	1.72***	1.44***	1.12
2013	1.57**	1.50***	0.53***	1.85***	1.76***	1.04
Interaction education x year						
No education						
2003	1.16	0.89	0.83	1.06	0.94	0.73**
2008	1.41**	0.90	0.87	1.25	0.94	0.83
2013	1.42	0.84	0.84	1.52*	0.60***	0.57*
Primary or incomplete secondary						
2003	1.05	0.95	0.91	0.84	0.98	0.76**
2008	1.23	0.95	0.88	0.96	0.96	0.85
2013	1.20	0.93	1.00	1.15	0.79	0.64
Secondary						
2003	1.14	0.75***	0.74*	0.94	0.92	0.65***
2008	1.52**	0.84*	0.89	1.05	0.91	0.76*
2013	1.22	0.93	0.97	1.17	0.95	0.61
Race (ref = white)						
Non-White	0.98	0.82***	0.95	0.98	0.89***	1.08
Proxy						
Yes	0.93*	0.85***	0.92**	0.98	0.93**	0.90**
Region (ref = Southeast)						
North	0.87*	0.74***	0.69***	0.78**	0.70***	0.69***
Northeast	0.83***	0.85***	0.61***	0.72***	0.75***	0.66***
Midwest	0.99	1.03	1.26***	0.89	0.93*	1.04
South	0.93	1.03	1.32***	0.84**	0.92*	1.13**
Health Insurance						
Yes	1.06	1.06*	1.10**	1.25***	1.14***	1.26***

*** *p* <0.001, ** *p* <0.01, * *p* <0.05

**Table 11:** Logistic regression results (log-odds) estimating the slope inequality index (SII) and its interaction with time for Diabetes, Hypertension and Heart Disease for the Total Population and by Sex, Brazil 1998–2013

Variables	All			Females			Males		
	Diabetes	Hypertension	Heart	Diabetes	Hypertension	Heart	Diabetes	Hypertension	Heart
Slope inequality index (SII)	−0.55***	−0.87***	−0.99***	−1.01***	−1.17***	−1.19***	0.24**	−0.42***	−0.68***
Year									
1998	0.00	0.00	0.00	0.00	0.00	0.00	0.00	0.00	0.00
2003	0.29***	0.14***	−0.16***	0.27***	0.16***	−0.17***	0.37***	0.12***	−0.13**
2008	0.54***	0.21***	−0.19***	0.49***	0.20***	−0.23***	0.72***	0.27***	−0.08
2013	0.88***	0.15***	−0.63***	0.80***	0.16***	−0.68***	1.05***	0.13**	−0.56***
Interaction SII x year									
2003	−0.18**	−0.08*	0.03	−0.23**	−0.13***	−0.00	−0.21*	−0.04	0.06
2008	−0.25***	−0.13***	−0.04	−0.33***	−0.19***	−0.10	−0.33***	−0.12*	−0.02
2013	−0.48***	0.16**	0.24	−0.50***	0.01	0.08	−0.59***	0.32***	0.39
Age (ref = age 25–49)									
50-94	1.73***	1.59***	1.54***	1.65***	1.60***	1.40***	1.81***	1.58***	1.73***
Race (ref = non-white)									
White	0.02	−0.11***	0.03	0.02	−0.16***	−0.02	0.01	−0.05**	0.10***
Proxy									
Yes	−0.07**	−0.15***	−0.14***	−0.05	−0.12***	−0.07**	−0.03	−0.07***	−0.11***
Region (ref = Southeast)									
North	−0.26***	−0.38***	−0.35***	−0.24***	−0.39***	−0.34***	−0.27***	−0.35***	−0.33***
Northeast	−0.32***	−0.24***	−0.48***	−0.26***	−0.21***	−0.51***	−0.38***	−0.27***	−0.41***
Midwest	−0.09**	−0.07***	0.07**	−0.04	−0.05**	0.11***	−0.14**	−0.06*	0.02
South	−0.11***	−0.03	0.15***	−0.06	−0.01	0.18***	−0.16**	−0.05	0.11**
Health Insurance									
Yes	0.17***	0.13***	0.18***	0.10**	0.05**	0.12***	0.25***	0.21***	0.23***

*** *p* <0.001, ** *p* <0.01, * *p* <0.05

## Abbreviations

CVD: Cardiovascular disease
PAHO: Panamerican Health Organization
PNAD: Brazilian National Household Survey (Pesquisa Nacional por Amostra de Domicílios)
PNS: Brazilian National Health Survey (Pesquisa Nacional de Saúde)
SII: Slope index of inequality
